# A Splice Defect in the *EDA* Gene in Dogs with an X-Linked Hypohidrotic Ectodermal Dysplasia (XLHED) Phenotype

**DOI:** 10.1534/g3.116.033225

**Published:** 2016-07-22

**Authors:** Dominik P. Waluk, Gila Zur, Ronnie Kaufmann, Monika M. Welle, Vidhya Jagannathan, Cord Drögemüller, Eliane J. Müller, Tosso Leeb, Arnaud Galichet

**Affiliations:** * Department of Clinical Research, Molecular Dermatology and Stem Cell Research, University of Bern, 3001, Switzerland; † DermFocus, University of Bern, 3001, Switzerland; § Institute of Animal Pathology, Vetsuisse Faculty, University of Bern, 3001, Switzerland; ** Institute of Genetics, Vetsuisse Faculty, University of Bern, 3001, Switzerland; †† Clinic for Dermatology, Inselspital, Bern University Hospital, University of Bern, 3001, Switzerland; ‡ Veterinary Teaching Hospital, The Koret School of Veterinary Medicine, The Hebrew University of Jerusalem, Rehovot, 7610001, Israel

**Keywords:** *Canis familiaris*, RNA-seq, splicing, X-chromosome, skin

## Abstract

X-linked hypohidrotic ectodermal dysplasia (XLHED) caused by variants in the *EDA* gene represents the most common ectodermal dysplasia in humans. We investigated three male mixed-breed dogs with an ectodermal dysplasia phenotype characterized by marked hypotrichosis and multifocal complete alopecia, almost complete absence of sweat and sebaceous glands, and altered dentition with missing and abnormally shaped teeth. Analysis of SNP chip genotypes and whole genome sequence data from the three affected dogs revealed that the affected dogs shared the same haplotype on a large segment of the X-chromosome, including the *EDA* gene. Unexpectedly, the whole genome sequence data did not reveal any nonsynonymous *EDA* variant in the affected dogs. We therefore performed an RNA-seq experiment on skin biopsies to search for changes in the transcriptome. This analysis revealed that the *EDA* transcript in the affected dogs lacked 103 nucleotides encoded by exon 2. We speculate that this exon skipping is caused by a genetic variant located in one of the large introns flanking this exon, which was missed by whole genome sequencing with the illumina short read technology. The altered *EDA* transcript splicing most likely causes the observed ectodermal dysplasia in the affected dogs. These dogs thus offer an excellent opportunity to gain insights into the complex splicing processes required for expression of the *EDA* gene, and other genes with large introns.

Ectodermal dysplasia (ED) is characterized by an abnormal development of several tissues and organs of ectodermal origin, including teeth, hair, nails, and sweat glands (Pinheiro and Freire-Maia 1994; Itin 2014). ED is genetically heterogeneous, and the most common form in humans is X-linked anhidrotic/hypohidrotic ectodermal dysplasia (XLHED) caused by variants in the *EDA* gene encoding ectodysplasin A (Kere *et al.* 1996; OMIM #305100). XLHED is clinically characterized by hypotrichosis, lacking and abnormally shaped teeth (oligodontia), and a lack of sweat glands and other eccrine glands (Pinheiro and Freire-Maia 1994) So far, > 100 independent *EDA* loss-of-function genetic variants have been described in human patients (*e.g.*, Huang *et al.* 2015; Ruiz-Heiland *et al.* 2016).

The *EDA* gene spans > 400 kb on the long arm of the X chromosome, and represents one of the largest genes in the human genome. It encodes several alternative transcripts and protein isoforms. The longest protein isoform (isoform 1) represents a 391-amino-acid type II transmembrane protein member of the tumor necrosis factor (TNF) superfamily, which is composed of a short intracellular domain, a transmembrane domain, a stalk region of uncharacterized function, a furin cleavage site required for proteolytic processing and release from the membrane, a short positively charged sequence mediating interactions with heparin-sulfate proteoglycans, a collagen domain involved in protein trimerization, and the C-terminal TNF homology domain responsible for receptor binding. Two distinct EDA receptors, EDAR and XEDAR, are known. EDA isoform 1 binds to EDAR, whereas XEDAR is activated by isoform 2 of the EDA protein (Hymowitz *et al.* 2003; Mikkola and Thesleff 2003). Activation of EDAR results in NFκB translocation to the nucleus, where it activates the transcription of various target genes required for the initiation and differentiation of ectodermally derived tissues such as hair, teeth, and sweat glands (Cui and Schlessinger 2006).

Spontaneous *EDA* mutants that can serve as animal models for the human disease have been described in mice (Srivastava *et al.* 1997), cattle (Drögemüller *et al.* 2001), and dogs (Casal *et al.* 2005). A research colony of dogs with a splice site variant affecting the last intron (*EDA*:c.910-1G>A) was established to develop therapeutic approaches. A single postnatal intravenous injection of recombinant soluble EDA protein led to a marked improvement of the dentition abnormalities in the permanent teeth of the treated dogs (Casal *et al.* 2007). Furthermore, normal lacrimation was restored, and the treated dogs were no longer prone to respiratory infections due to restoration of their mucous glands and improved mucociliary clearance in the tracheal and bronchial airway epithelia (Mauldin *et al.* 2009).

In this study, we describe the phenotype of canine XLHED in mixed-breed dogs from Israel. We applied whole genome resequencing (WGS) and global transcriptome analyses (RNA-seq) to unravel the molecular aberrations causing this phenotype.

## Materials and Methods

### Ethics statement

All animal experiments were performed according to the local regulations. All dogs in this study were privately owned and examined with the consent of their owners. The study was approved by the “Cantonal Committee For Animal Experiments” (Canton of Bern; permit 23/10).

### Skin biopsy sampling and histopathology

Six millimeter punch biopsies were obtained from the skin of XLHED affected dogs after sedation and local anesthesia with 2% lidocaine. The biopsies were collected from alopecic and hypotrichotic areas of the skin. Biopsies were either fixed in 10% buffered formalin, routinely processed, and stained with hematoxylin and eosin prior to histopathological examination, or immersed in RNAlater for RNA extraction.

### DNA samples and SNP genotyping

We used three XLHED affected dogs (DS032, DS041, and DS042) for the genetic analysis. Genomic DNA from EDTA blood samples was isolated with either the Nucleon Bacc2 kit (GE Healthcare), or a Maxwell RSC automated DNA extraction instrument (Promega). Genotyping was done on illumina canine_HD chips containing 173,662 SNPs by GeneSeek/Neogen. Genotypes were stored in a BC/Gene database, version 3.5 (BC/Platforms).

### Identical by descent estimation and homozygosity mapping

PLINK v1.07 (Purcell *et al.* 2007), with the “–genome” option, was used to calculate the proportion of identical by state (IBS) genotypes at the 173,662 markers from the illumina canine_HD chip. Given the large number of markers, this yields an accurate genome-wide estimation of the proportion of pairwise identical by descent (IBD) segments.

The options “–homozyg” and “–homozyg–group” were applied to search for extended intervals of homozygosity with shared alleles across the three affected animals. Using these standard parameters, PLINK reports homozygous segments ≥ 1 Mb.

### Whole genome sequencing of the three affected dogs

We prepared illumina PCR-free fragment libraries with 350 bp insert sizes. The library of dog DS032 was sequenced to 14× coverage on an illumina HiSeq2500 instrument using 2 × 100 bp paired-end reads. The libraries of the dogs DS041 and DS042 were sequenced with 2 × 150 bp reads to 28× and 27× coverage on a HiSeq3000 instrument. The mapping and variant calling was done as described previously (Drögemüller *et al.* 2014). The reads obtained from whole genome sequencing were also visualized in the Integrated Genome Viewer (Broad Institute).

### RNA-seq from skin biopsies

We isolated total RNA from skin biopsies from two of the affected dogs (DS032 (1 sample from a hypotrichotic area, DS032_H) and DS042 (2 samples from alopecic and hypotrichotic areas, DS042_A and DS042_H)) using the RNeasy Fibrous Tissue Mini kit (Qiagen). Prior to RNA extraction, the tissue was mechanically disrupted using the TissueRuptor device (Qiagen). The RNA samples were transformed into illumina TruSeq libraries and 2 × 150 bp sequencing reads were obtained on a HiSeq3000 instrument (Illumina). We additionally used publicly available skin RNA-seq datasets from the skin of two dogs, a Beagle, and a dog of unspecified breed (accession PRJNA78827; Hoeppner *et al.* 2014).

### RNA-seq data analysis

For each affected dog sample, 43–50 million paired-end reads were produced. The control dog datasets “skin_begl” and “SRR536884” consisted of 43 million and 18 million paired-end reads, respectively. The reads were mapped to the CanFam3.1 dog reference genome assembly using STAR (Dobin *et al.* 2013) (version STAR_2.4.0g using default parameters; multi hits at N50). Read counts were generated by HTSeq (v0.5.3p9, Anders *et al.* 2015) against Ensembl v74 gene annotations. The counts were processed in DeSeq2 (v1.10.1 Love *et al.* 2014) built with the R statistical software, version 3.2.3 using geometric mean normalization of DeSeq2, paired design, and estimation of differential expression using a generalized linear model. The fold change of logarithm of base 2 of normalized data (logFC) was used to rank the data from top upregulated to top downregulated genes, and a Bonferroni-adjusted P-value < 0.05 was used to define significantly differentially expressed genes. Sequence quality metrics were assessed using FastQC (http://www.bioinformatics.babraham.ac.uk/projects/fastqc/). Pathway analysis was carried out using MetaCore from GeneGo (Thomson Reuters software).

### Genome reference assembly and gene reference

We used the dog CanFam 3.1 assembly for all analyses. Numbering within the canine *EDA* gene corresponds to the accession NM_001014770.2 (mRNA).

### RT-PCR

Reverse transcription reactions from the RNA samples of two affected dogs, and one control Beagle dog, were performed using Super Script IV Reverse Transcriptase (Invitrogen, LifeTechnologies). We then amplified a product containing the entire open reading frame of the *EDA* transcript, using a forward primer in exon 1 (GCCTCAGAGAGTGGGTGTCT), and a reverse primer in exon 8 (CCTGGAGTCACTGGGGAATA). For the RT-PCR, 1 µl of cDNA and 25 pmol of each primer were used in a 50 µl reaction with ATG360 polymerase and 360GC Enhancer according to the manufacturer’s specifications (Invitrogen, LifeTechnologies). Cycling conditions consisted of 10 min initial denaturation at 95°, followed by 35 cycles of 30 sec at 95°, 30 sec at 60°, 2 min at 72°, and a final extension step of 7 min at 72°. The resulting RT-PCR products were analyzed by direct Sanger sequencing (see below). For the visualization of the splice defect, we used 1 µl of the RT-PCR reactions, and reamplified a smaller product using the nested primers CTAGAGTTGCGCTCCGAGTT and TCCTGCCTTCTTTCCCTTTT and PCR conditions as described above. The size of the RT-PCR products was determined on the Fragment Analyzer capillary gel electrophoresis instrument (Advanced Analytical).

### Sanger sequencing

We used Sanger sequencing to confirm the splice defect identified in the illumina RNA-seq data, and to verify the identity of RT-PCR products. RT-PCR products were sequenced directly on an ABI 3730 capillary sequencer (LifeTechnologies) after treatment with exonuclease I and shrimp alkaline phosphatase. We analyzed the Sanger sequence data with Sequencher 5.1 (GeneCodes).

### Data availability

Supplemental Material, Figure S1 illustrates the Sanger sequencing data confirming the *EDA* splice defect. Figure S2 shows an alignment of the wildtype canine EDA protein sequence with the predicted mutant protein from the transcript in XLHED affected dogs. Table S1 summarizes the results from the individual clinical examinations. Table S2 lists the results from the transcriptome analysis in the skin of XLHED affected dogs. Table S3 summarizes the result from the pathway analysis of the RNA-seq data. The whole genome sequencing data were deposited under accessions PRJEB14110 (DS032, DS041, and DS042), and the RNA-seq data (DS032_H, DS042_A, DS042_H) under accession PRJEB14109 at the European Nucleotide Archive.

## Results

### Clinical presentation and laboratory findings

**Figure 1:**
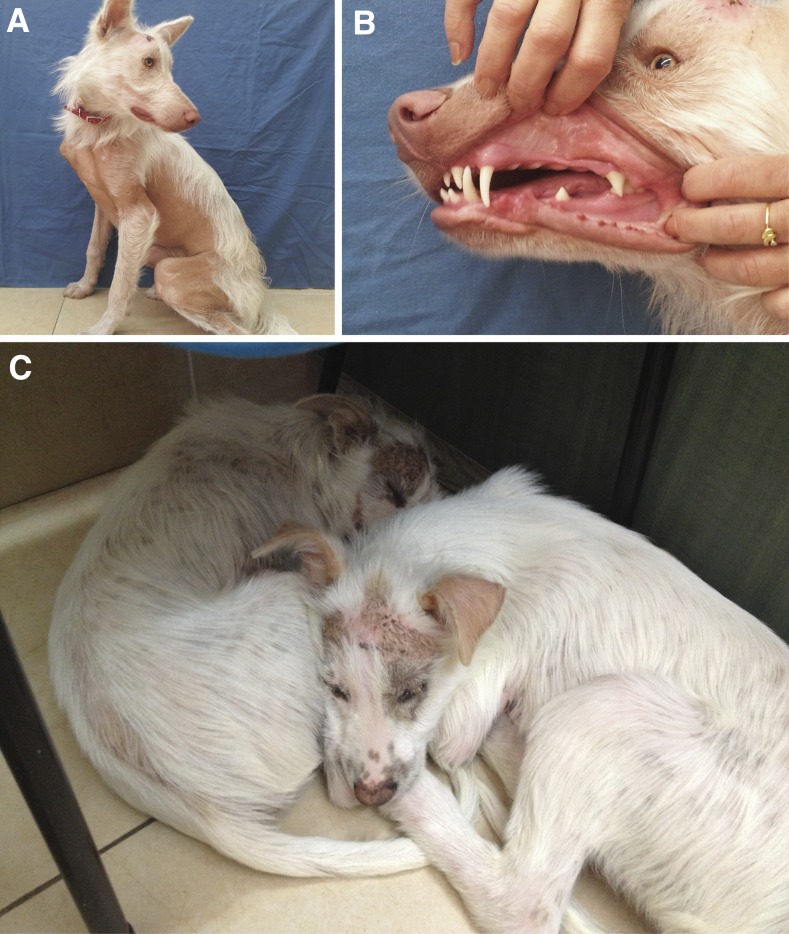
Ectodermal dysplasia phenotype. (A) Pronounced hypotrichosis and alopecia in an affected dog (DS032) at 14 months of age. (B) Dentition abnormalities in the same dog. Note several missing molars in the upper and lower jaw, and the pointed shape of the existing incisors and molars. (C) Two 3-month-old brothers (DS041 and DS042) with unknown familial relationship to the first case also presented with pronounced hypotrichosis, and some completely hairless skin areas.

The first of three XLHED cases was diagnosed in 2012. This was a 14-month-old mixed-breed male dog (DS032) that was presented to the dermatology department at the Veterinary Teaching Hospital of the Koret School of Veterinary Medicine, The Hebrew University of Jerusalem, with the chief complaint of ulcerative lesions on the skin of the frontotemporal region. The dog had been adopted at the age of 2 months by a rescue organization, and the adopters reported that one of its littermates had died at a few weeks of age. Both dogs showed spreading hair loss. The adopted dog was completely bald on the fronto-temporal region, the proximal limbs, the entire ventral abdomen and chest, and had sparse hair-coat on his back (Figure 1A). The hair-loss did not change over the 1 yr period since adoption, and the ulcerative skin lesions had appeared a few weeks prior to presentation. On presentation, abnormal dentition was recognized: absence of premolar and some incisors, as well as conical shape of incisors, canine, and molar teeth (Figure 1B).

In 2014, two 3-month-old mixed-breed brothers were presented from another rescue organization (DS041 and DS042). These dogs had a similar pattern of hair loss, predominantly on the fronto-temporal region and the ventral part of the body. Interestingly, these dogs were found in the same part of the country as the first one; however, no familial relationship could be established. There was a mild cocoid bacterial infection, and no parasites or dermatophytes were detected. After a few months, the dogs were adopted and 1 yr later, at the age of 1.5 yr, both of them returned for recheck. In addition to the alopecia that had been described at puppyhood, and had progressed, the dogs also had the typical dentition abnormalities described for XLHED (Casal *et al.* 1997). Opthalmoscopic examination and Schirmer tear tests were normal. All three dogs were otherwise healthy, and no respiratory or ophthalmic abnormalities were noticed. The clinical findings are summarized in Table S1.

### Histopathological findings

**Figure 2:**
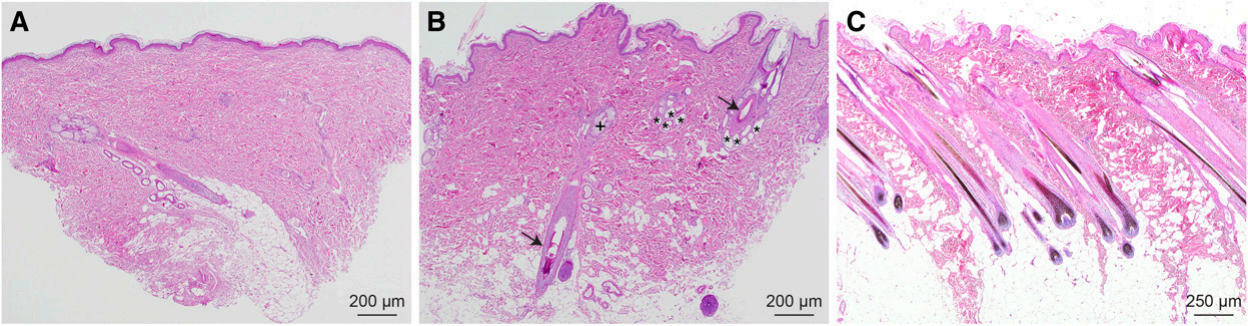
Histopathology of a dog with ectodermal dysplasia. (A) Image taken from an alopecic area. Only one simple hair follicle with its associated sebaceous and sweat glands is present within the entire dermis. Stained with hematoxylin & eosin. (B) Image taken from a hypotrichotic area. Hair follicles are distributed regularly within the dermis. These are also simple follicles, and, only rarely, two follicles are neighboring each other. Stained with hematoxylin & eosin. (C) Skin biopsy of a normal dog. The hair follicles are grouped together forming compounds composed of several hair follicles, sweat glands, and sebaceous glands. Stained with hematoxylin & eosin. The arrows in (B) depict hair follicles, the stars indicate sweat glands, and the plus sign indicates sebaceous glands.

The histopathological findings were similar in all skin biopsies taken from the three XLHED affected dogs. In the alopecic skin, the vast majority of adnexal structures, including hair follicles, sebaceous glands, and sweat glands were missing in the otherwise normal dermis. Very rarely, isolated simple hair follicles with associated sebaceous and sweat glands were present (Figure 2A). In skin biopsies taken from hypotrichotic skin of the affected dogs, hair follicles, and the associated sebaceous and sweat glands were present within the entire dermis, but these hair follicles also represented simple follicles, and, only very rarely, two follicles were neighboring each other (Figure 2B). Dogs normally have compound hair follicles composed of up to 30 hair follicles within one follicular compound. In the skin biopsies of one of the XLHED dogs, additional sun-induced changes such as solar elastosis, vasculopathy, and a UV-induced hemangiosarcoma were present.

### Genetic mapping of the causative genetic defect

The affected dogs were mixed-breed dogs and did not have official pedigree certificates. Two of the affected dogs were brothers and the relation to the third dog was unknown. We initially genotyped the dogs on illumina SNP arrays containing 173,662 markers. The SNP data confirmed that two of our sampled dogs were full brothers as 53% of their genome was estimated to be identical by descent (IBD). In contrast, the third affected dog was estimated to share only 2% and 3% IBD regions with the two brothers and thus was probably not extremely closely related to these dogs.

We hypothesized that a recessive mode of inheritance would be most likely, and therefore then searched for shared homozygous genome segments among the three cases. This analysis revealed a large shared haplotype of 51 Mb on the X-chromosome, but no shared extended homozygous regions on the autosomes. Thus, the SNP data were in agreement with a monogenic X-linked recessive mode of inheritance for the ectodermal dysplasia.

In order to identify the causative genetic variant, we sequenced the genomes of all three cases at 14× – 28× average coverage, which equals to 7× – 14× average coverage of the X-chromosome. The sequencing data confirmed and refined the shared haplotype, which spanned from 17,759,715 – 68,640,703 bp on the X-chromosome (CanFam 3.1 assembly).

We called single nucleotide and small indel variants in the three affected dogs and compared them to the reference genome derived from a nonaffected Boxer. All exons and all splice sites of the *EDA* gene were normal and did not reveal any likely candidate causative genetic variants for the observed XLHED phenotype.

### Transcriptome analysis

As the whole genomic sequencing experiments had not revealed any good candidate causative variants, we isolated RNA from skin biopsies and performed an mRNA-seq experiment. Three RNA samples from two affected dogs and two RNA samples from two control dogs were available for this experiment.

In the affected dogs, 22% of the genes were upregulated, and 26% were downregulated (Table S2). The *EDA* expression level in the affected dogs was 2.12-fold lower compared to the controls (P_adj_ = 0.002). Interestingly, many differentially expressed genes belonged to the Wnt, transforming growth factor-β (TGFβ), and TNF signaling pathways (Table S3). The role of the TGFβ and Wnt pathways downstream of EDA was previously reported (Cui *et al.* 2006; Mou *et al.* 2006; Zhang *et al.* 2009). Additionally, our data analysis revealed transcriptional regulation of genes involved in tooth development, such as *PAX9* and *MSX1* (Peters *et al.* 1998; Satokata and Maas 1994).

### A splice defect in EDA

The *EDA* gene represents the primary functional candidate gene for the observed ectodermal dysplasia phenotype, and is located at 54,078,743–54,515,535 bp on the X-chromosome within the shared haplotype on the X-chromosome. *EDA* is expressed at low levels in adult skin. We therefore also visually inspected the *EDA* transcript in the RNA-seq data using the IGV browser, and discovered that exon 2 was missing from the transcripts of affected dogs, while it was unremarkable in control dogs.

**Figure 3:**
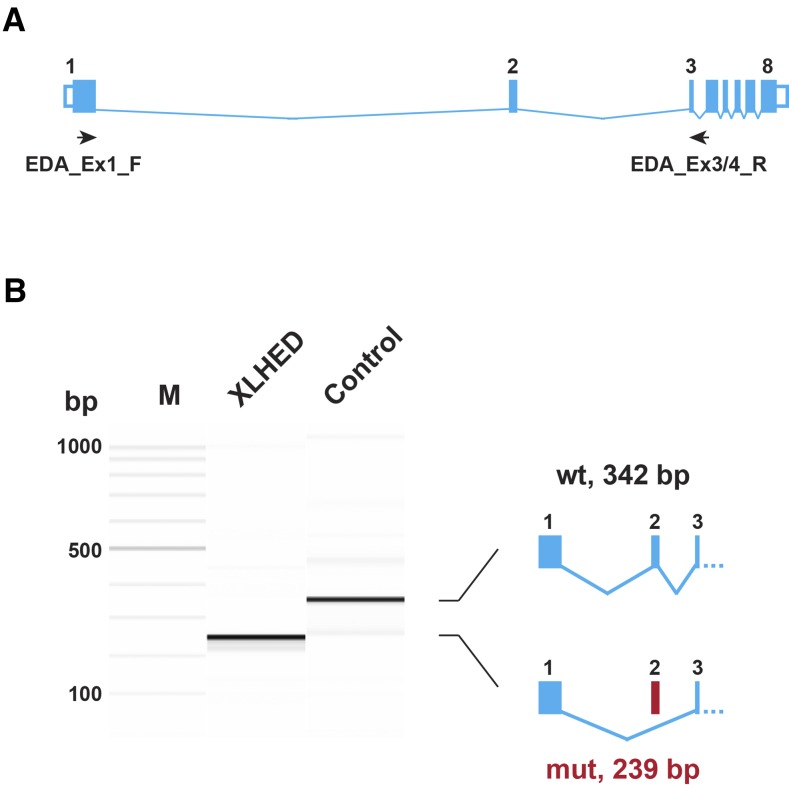
RT-PCR confirmation of the splice defect. (A) Genomic organization of the canine *EDA* gene with its eight exons. The positions of a forward primer in exon 1, and a reverse primer at the boundary of exon 3 and exon 4, for the RT-PCR experiment are indicated. (B) RT-PCR was performed using RNA from skin biopsies of an XLHED-affected and a control dog. The picture shows a Fragment Analyzer gel image of the experiment. In the control animal, the expected 342 bp band is visible. In the XLHED-affected dog, the experiment resulted in a 239-bp band corresponding to a transcript lacking exon 2. The identity of the bands was confirmed by Sanger sequencing (Figure S1).

We designed primers for RT-PCR, and confirmed the absence of exon 2 from the *EDA* transcripts of XLHED-affected dogs in an independent experiment (Figure 3 and Figure S1). The variant designation for this frame-shifting exon skipping on the transcript level is r.385_487del. The predicted variant on the protein level is p.Met129Valfs*112, and the predicted mutant protein lacks the functionally important collagen-like and TNF-signaling domains (Figure S2).

## Discussion

In this study, we identified a novel splice defect of the *EDA* gene as the likely cause of an ectodermal dysplasia in dogs. Interestingly, the aberrant transcript in the XLHED-affected dogs corresponded exactly to the mutant transcript in previously reported cattle with an ectodermal dysplasia. However, in the mutant cattle, the loss of exon 2 was caused by a large genomic deletion (Drögemüller *et al.* 2001).

While we found clear evidence for the splice defect in the XLHED dogs on the RNA level, we were unable to identify the underlying causative sequence variation on the genomic DNA level. This study highlights the limitations of current sequencing technologies. Short-read exome or WGS is presently the method of choice to identify genetic variants in human and animal patients with suspected inherited diseases. While there are numerous examples where this approach can be applied successfully, it failed in this project. Despite having fairly high whole genome sequence coverage at close to 30× in two of our cases, we were unable to identify any private genomic sequence variant within the *EDA* gene. This leaves two possibilities: (1) the genetic variant causing the splice defect might be located outside of the *EDA* gene. We consider this scenario as unlikely, but it should not be categorically disregarded until the causative genetic variant has been definitively identified. (2) The causative genetic variant might be located in a gap of the reference genome, or in one of the repetitive regions within the *EDA* gene, where the short 2 × 150 bp illumina reads do not map unambiguously. We consider this as the more likely scenario. It has to be re-emphasized that the genomic sequences of exon 2 and the consensus splice sites flanking introns 1 and 2 were normal in the affected dogs (Figure S1). Thus, the causative genetic defect is most likely located in regions of the gene that are normally not considered to be essential for splicing.

Exon 2 of the *EDA* gene may represent the “loneliest” exon in the mammalian genome. Its flanking introns are larger than 300 kb and 100 kb in size, respectively. During transcription, the RNA polymerase will have rotated 30,000 times around the template DNA strand, and the primary transcript will have reached a length of ∼100 µm before the first intron can be spliced by the spliceosome. Thus, recognition of the correct splice sites in the *EDA* transcript is an astonishing feat of the splicing machinery, and may very well depend on additional noncanonical intronic sequence elements and accessory splicing factors that ensure the correct spatial arrangement of the 5′- and 3′-splice sites in the primary transcript. Alternatively, spliceosomal RNAs which assist in fine tuning spliceosomes might be differentially modified by small RNAs encoded in intron 1 or 2 of the affected dogs (Patil *et al.* 2015).

Two other examples from cattle with XLHED underscore some peculiarities of splicing defects in the *EDA* gene: in an affected calf carrying a single nucleotide variant located in the 5′-splice site of intron 8 (c.924+2G>T), not only the splicing of intron 8, but unexpectedly also the splicing of intron 7 was found to be altered (Drögemüller *et al.* 2002). In another XLHED affected calf, *EDA* transcripts were found to contain an extra 161 nucleotides, presumably derived by aberrant “exonization” of a LINE element inserted into intron 1 of the *EDA* gene (Karlskov-Mortensen *et al.* 2011). These examples show that genomic sequence changes in the *EDA* gene can result in unexpected splice defects and XLHED.

Our hypothesis of an unidentified genetic variant within the *EDA* gene as the cause for exon skipping in the dogs with ectodermal dysplasia might be proven in the future; the availability of better sequencing technologies with longer read lengths that enable *de novo* assemblies and the possibility to reliably sequence long interspersed repeats should make it possible to identify the elusive genetic variant.

In conclusion, we provide a phenotypic description of dogs with an ectodermal dysplasia phenotype, and identified an unusual splice defect of the *EDA* gene, which most likely causes this phenotype. These dogs thus offer an excellent opportunity to gain insight about the complex splicing processes in the *EDA* gene, and possibly also other genes with very large introns.

## Supplementary Material

Supplemental Material
