# Supplementary material for: A Splice Defect in the EDA Gene in Dogs with an X-Linked Hypohidrotic Ectodermal Dysplasia (XLHED) Phenotype
Source: G3 (Bethesda). 2016 Jul 22;6(9):2949–54. doi: 10.1534/g3.116.033225 (PMC5015951; doi:10.1534/g3.116.033225)
Supplement: Supplemental Material [file supp_6_9_2949__index.html]

A Splice Defect in the EDA Gene in Dogs with an X-Linked Hypohidrotic Ectodermal Dysplasia (XLHED) Phenotype — Supplemental Material 

# A Splice Defect in the *EDA* Gene in Dogs with an X-Linked Hypohidrotic Ectodermal Dysplasia (XLHED) Phenotype

## Supplemental Material for Waluk, *et al*, 2016

**Files in this Data Supplement:**

- Figure S1 - Sanger sequencing confirmation of the EDA splice defect. (.pdf, 334 KB)
- Figure S2 - Alignment of the wildtype canine EDA protein sequence with the predicted mutant protein from the transcript in XLHED affected dogs (p.Met129Valfs\*112). (.pdf, 45 KB)
- Table S1 - Results of the individual clinical examinations of the three affected dogs. (.xlsx, 14 KB)
- Table S2 - Results from the transcriptome analysis. (.xlsx, 2179 KB)
- Table S3 - Top 10 results from pathway analysis using MetaCoreTM from GeneGo (Thomson Reuters software). (.xlsx, 15 KB)
