# Supplementary material for: A Splice Defect in the EDA Gene in Dogs with an X-Linked Hypohidrotic Ectodermal Dysplasia (XLHED) Phenotype
Source: G3 (Bethesda). 2016 Jul 22;6(9):2949–54. doi: 10.1534/g3.116.033225 (PMC5015951; doi:10.1534/g3.116.033225)
Supplement: Supplemental Material [file supp_g3.116.033225_FigureS1.pdf]

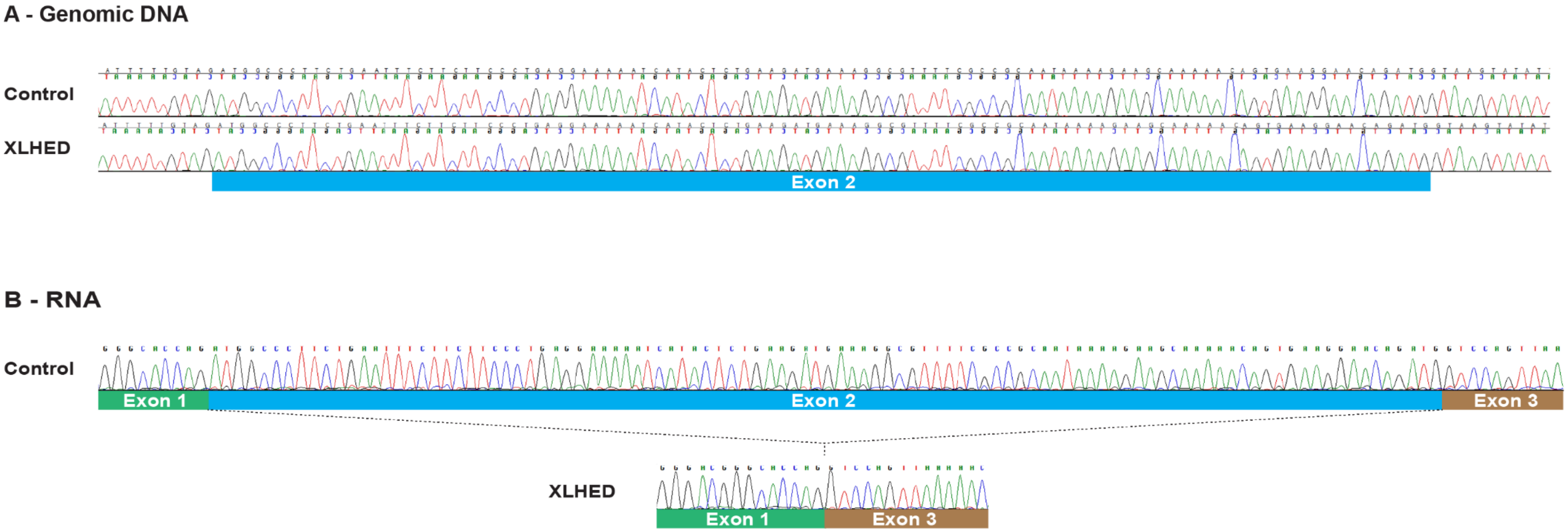

Supplementary Figure 1 Sanger sequencing confirmation of the *EDA* splice defect. (**A**) Genomic sequences from a control and an XLHED affected dog in the region of exon 2 of the *EDA* gene. The sequences are identical between the two animals and also identical to the CanFam 3.1 reference genome assembly. (**B**) Sequences of RT-PCR products derived from skin RNA. The control animal shows the expected transcript, while in the XLHED affected dog the 103 nucleotides encoded by exon 2 are missing.
