# Supplementary material for: A Splice Defect in the EDA Gene in Dogs with an X-Linked Hypohidrotic Ectodermal Dysplasia (XLHED) Phenotype
Source: G3 (Bethesda). 2016 Jul 22;6(9):2949–54. doi: 10.1534/g3.116.033225 (PMC5015951; doi:10.1534/g3.116.033225)
Supplement: Supplemental Material [file supp_g3.116.033225_FigureS2.pdf]

```
wildtype   MGYPEVERRE PLPTAAPRER GSQGCGCRGA PAQAGEGNSC RLFLGFFGLS    50
XLHED      MGYPEVERRE PLPTAAPRER GSQGCGCRGA PAQAGEGNSC RLFLGFFGLS    50

wildtype   LALHLLTLCC YLELRSELRR ERGAESRLGP GTPGTLNSPG GLDPDGPITR   100
XLHED      LALHLLTLCC YLELRSELRR ERGAESRLGP GTPGTLNSPG GLDPDGPITR   100

wildtype   DSGQPSPQQQ PLEAEETALP PHSRDGHQMA LLNFFFPEEK SYSEDERRFR   150
XLHED      DSGQPSPQQQ PLEAEETALP PHSRDGHQVQ LKTRKRERRQ DLLGPMALQV   150

wildtype   RNKRSKNSEG TDGPVKNKKK GKKAGPPGPN GPPGPPGPPG PQGPPGIPGI   200
XLHED      LQALQDPRDP QGFQEFLGFQ EQLLWDHLAL QVLLVLRDPL AYRDLLVLLI   200

wildtype   PGIPGTTVMG PPGPPGPPGP QGPPGLQGPS GTADKAGPRE NQPAVVHLQG   250
XLHED      KLDLEKTSQL WCIYRAKGQQ FKSRMIFQVE CSMTGLASP*              239

wildtype   QGSAIQVKND LSGGVLNDWS RITMNPKVFK LHPRSGELEV LVDGTYFIYS   300

wildtype   QVEVYYINFT DFASYEVVVD EKPFLQCTRS IETGKTNYNT CYTAGVCLLK   350

wildtype   ARQKIAVKMV HADISINMSK HTTFFGAIRL GEAPAS                  386
```

Supplementary Figure 2 Alignment of the wildtype canine EDA protein sequence with the predicted mutant protein from the transcript in XLHED affected dogs (p.Met129Valfs*112). Skipping of exon 2 in the mutant *EDA* transcript causes a frame shift and results in a premature stop codon and the truncation of the functionally important collagen-like and TNF-signaling domains.
